# Maternal Uniparental Isodisomy of Chromosome 6: A Novel Case of Teratoma and Autism Spectrum Disorder with a Diagnostic and Management Framework

**DOI:** 10.3390/genes16040434

**Published:** 2025-04-05

**Authors:** Aleksandra Świeca, Maria Franaszczyk, Agnieszka Maryniak, Patryk Lipiński, Rafał Płoski, Krzysztof Szczałuba

**Affiliations:** 1 Department of Medical Genetics, Medical University of Warsaw, 02-106 Warsaw, Poland; 2 The Faculty of Psychology, University of Warsaw, 00-183 Warsaw, Poland; 3 Institute of Clinical Sciences, Maria Skłodowska-Curie Medical Academy, 00-136 Warsaw, Poland; 4 Department of Pediatrics, Bielański Hospital, 01-809 Warsaw, Poland; 5 Center of Excellence for Rare and Undiagnosed Disorders, Medical University of Warsaw, 02-106 Warsaw, Poland

**Keywords:** uniparental disomy, chromosomes, human, pair 6, fetal growth retardation, teratoma, autism spectrum disorder, imprinting disorders, intellectual disability

## Abstract

Background: Uniparental disomy (UPD) is the inheritance of both copies of a chromosome from a single parent, leading to distinct genetic conditions. Maternal UPD of chromosome 6 (UPD(6)mat) is extremely rare, with few molecularly confirmed cases reported. Methods: We report a prematurely born female with isodisomic UPD(6)mat, presenting with intrauterine growth restriction (IUGR), developmental delay, autism spectrum disorder, dysmorphic features, and a sacrococcygeal teratoma. In addition, we reviewed 24 confirmed UPD(6)mat cases to assess clinical patterns in prenatal findings, birth outcomes, and postnatal features. Results: Trio whole-exome sequencing revealed complete isodisomy of chromosome 6 and a de novo heterozygous *DIAPH2* variant of uncertain significance. In the literature review, IUGR was present in 87% of cases, with most individuals born small for gestational age and preterm. Failure to thrive and neurodevelopmental issues were also frequent. While the exact molecular basis remains unknown, imprinting disturbances—similar to those in UPD(6)pat—and cryptic trisomy 6 mosaicism, particularly in heterodisomy, are the most likely mechanisms. No specific gene or consistent epigenetic abnormality has been identified. Conclusions: This study aims to enhance the understanding of the genetic and phenotypic spectrum of UPD(6)mat, improving diagnostic and management approaches for this ultra-rare genetic disorder. We propose a detailed list of clinical assessments and tests to be performed following the detection of maternal uniparental disomy of chromosome 6.

## 1. Introduction

Uniparental disomy (UPD) occurs when both homologous chromosomes are derived from a single parent, with none originating from the other parent. The phenotypic abnormal outcomes of UPD fundamentally depend on the parent of origin, necessitating differentiation between maternal (UPDmat) and paternal (UPDpat) uniparental disomy. UPDmat is observed to occur twice as frequently as the less common UPDpat [1]. The concept of UPD was introduced by Engel in 1980, with the first case reported in 1988 involving a patient with cystic fibrosis and two maternal copies of chromosome 7 [2,3]. Initially considered rare, later reports suggested UPD may be more common. Current estimates indicate a prevalence of 1 in 2000 individuals [4]. Although UPD typically does not result in phenotypic effects in most cases, imprinted regions on certain chromosomes can lead to notable clinical outcomes [5]. UPD has been implicated in several well-known imprinting disorders, including Prader–Willi syndrome (UPD(15)mat), Angelman syndrome (UPD(15)pat), and transient neonatal diabetes mellitus (UPD(6)pat). UPD can manifest as isodisomy (two identical chromosomes from one parent) or heterodisomy (two homologous chromosomes from one parent). Segmental isodisomy results in partial copy-neutral loss of heterozygosity, a phenomenon that plays a significant role in cancer development. UPD(6)pat, leading to overexpression of the imprinted genes *PLAGL1* and *HYMAI*, has been linked to intrauterine growth restriction (IUGR), transient neonatal diabetes mellitus, and a range of other clinical features [6]. UPD(6)mat is generally associated with IUGR and preterm birth, with no consistent phenotype–genotype correlation identified. Furthermore, the mechanism underlying these clinical features remains unknown.

In this study, we present our unique case of a female patient with sacrococcygeal teratoma and autistic features—the phenotype not previously associated with this ultra-rare genetic condition (Table 1). We review the literature on UPD(6)mat and its potential role as an imprinting disorder and examine imprinted genes located on chromosome 6. Based on these findings, we propose a diagnostic and management framework to guide clinicians in managing UPD(6)mat cases.

## 2. Materials and Methods

DNA from the proband and her parents was extracted from peripheral blood leukocytes with a standard automated protocol. WES (whole exome sequencing) library preparation for the trio (Trio WES) was performed with Twist Exome 2.0 blended and spiked-in with Twist mtDNA Panel (Twist Bioscience, San Francisco, CA, USA). The enriched library was paired-end sequenced (2× 100 bp) on NovaSeq 6000 (Illumina, San Diego, CA, USA) with a final 124,345,293 reads resulting in a mean depth of 116.18× (20× coverage of target bases was 99.5% and 10× was 99.6%). Reads were aligned to the GRCh38 (hg38). Informed consent was obtained from the proband’s parents for their participation in this study, including permission to publish clinical data and identifying photographs.

## 3. Results

### 3.1. Case Report

A female preterm infant, born at 35 weeks via Caesarean section due to perinatal asphyxia and sacrococcygeal teratoma, was the first child of non-consanguineous parents. The pregnancy was complicated by polyhydramnios, managed through amniotic fluid drainage, and sacrococcygeal teratoma, treated with five in utero shunt procedures. At birth, the infant weighed 1450 g (−2.7 SD), measured 43 cm (−1.1 SD), and had a head circumference of 30 cm (−1.2 SD) [8]. Six days postnatally, the tumor was excised, with the sacrum preserved. No additional congenital anomalies were noted. Feeding difficulties led to a neurologopedic evaluation at 15 days, revealing an enhanced bite reflex. Developmental delays included sitting unassisted at 10 months, walking at 18 months, and first words at 18 months. Hearing was normal, but speech remained delayed, progressing to forming simple sentences by age 2. At 3 years and 2 months, she contracted a severe respiratory infection, with negative tests for Epstein–Barr virus and SARS-CoV-2. This infection led to cognitive regression, and she did not recover her pre-infection abilities. A head MRI and electroencephalography at 3.5 years showed no abnormalities, and immunological tests were normal, including negative results for anti-cerebellar and anti-Borrelia antibodies. Despite cognitive therapy, she stopped pointing at objects and ceased speaking. Growth delay was confirmed, though the growth hormone secretion pattern was normal.

**Figure 1:**
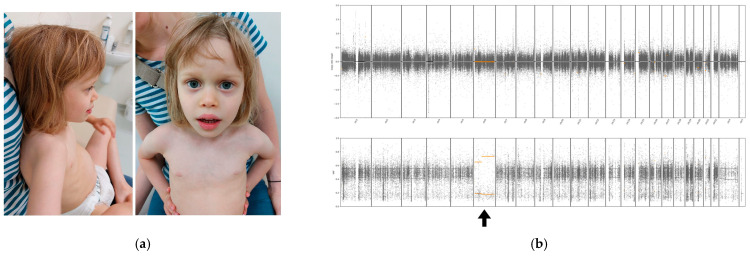
(**a**) Clinical features at age 4 years 10 months. Generalized deficiency of subcutaneous fat can be seen. The face is triangular with a prominent forehead, slightly upturned nose, and full lips; (**b**) Arrow showing region of homozygosity spanning the entire chromosome 6 in the proband.

At 4 years and 10 months, she was referred to the Genetics Clinic, where significant delays in receptive and expressive language were observed, with vocabulary limited to single words. She exhibited sensory hypersensitivity, sleep disturbances, and Autism spectrum disorder (ASD)-like behaviors, including mouthing objects. Her growth parameters were below the 3rd percentile, and she displayed distinct facial features (triangular face, prominent forehead, full lips) (Figure 1a). Trio Whole-Exome Sequencing (TrioWES) was performed. At 5 years and 3 months, neuropsychological evaluation revealed a short attention span and echolalia. She fatigued easily with environmental changes, and adaptive behavior assessments indicated extremely low functioning across all scales. Autism Spectrum Rating Scales (ASRS) scores were consistent with ASD, particularly in social communication. By 5 years and 8 months, metabolic evaluations, including tests for creatine kinase, urea, and acylcarnitine profiles, were normal. Given the presence of teeth grinding and distinctive hand movements, *MECP2* gene reanalysis was conducted, confirming the absence of mutations, and repeated brain MRI showed no abnormalities.

### 3.2. Genetic Analysis

The zygosity analysis of the variants identified a region of homozygosity spanning the entire chromosome 6 in the proband (Figure 1b). Further verification and assessment of the parental origin of these variants revealed uniparental maternal isodisomy of chromosome 6. Our patient was also identified with a heterozygous de novo *DIAPH2* gene variant at Xq21.33. With limited information available, *DIAPH2* is known to potentially play a role in oogenesis, and its variant may be associated with Premature Ovarian Failure 2A and amenorrhea [9]. No direct link has been established between the *DIAPH2* variant and the symptoms presented in our case. No other genetic variants that could contribute to the proband’s phenotype (including no pathogenic/likely pathogenic homozygous variants) were identified.

## 4. Discussion

UPD(6)mat remains an exceptionally rare and poorly characterized condition, with few confirmed cases and limited long-term follow-up. In this study, we reviewed previously published cases alongside our own to identify recurring features and potential pathogenic mechanisms. Given the suspected role of imprinting in UPD-related disorders, we examined candidate imprinted genes on chromosome 6 that may contribute to the phenotype. Our case included a sacrococcygeal teratoma detected prenatally—an uncommon finding not previously reported in UPD(6)mat. This prompted a broader review to evaluate whether neoplasms occur as part of UPD or are incidental findings. Based on our analysis, we propose practical considerations for diagnosis, monitoring, and follow-up in affected individuals.

### 4.1. Literature Review of UPD(6) Cases

A structured literature search was performed on 23 March 2025 in PubMed, Embase, and Scopus using the query: (“uniparental disomy”[MeSH] OR “uniparental disomy” OR “UPD”) AND (“maternal” OR “maternal origin” OR “UPD(6)mat”) AND (“chromosome 6”). The search yielded 148 articles (Embase: 65; PubMed: 29; Scopus: 54). After removing duplicates, 70 unique articles remained, and 19 met inclusion criteria: confirmed UPD(6)mat and available clinical data. We excluded studies on UPD(6)pat, unconfirmed cases, animal studies, and reviews lacking patient-level detail. Including our case, we analyzed 24 confirmed UPD(6)mat cases and extracted clinical data on UPD subtype, sex, presence of monogenic mutations, variant interpretation, IUGR, additional prenatal findings, gestational age at birth, birth weight, weight z-score, birth weight classification, failure to thrive (FTT), and postnatal phenotypic features [8].

We aimed to explore phenotypic patterns associated with different types of UPD(6)mat. In our reviewed cohort, isodisomy was more frequently linked with homozygous monogenic variants, consistent with maternal inheritance of recessive alleles. In contrast, heterodisomy and mixed hetero/isodisomy cases were more often associated with placental findings, including confined placental mosaicism and evidence of trisomy 6 mosaicism. Across all cases, intrauterine growth restriction (IUGR) was observed in 87%, and 71% of neonates were small for gestational age (SGA). The mean birth weight was 1503.5 g, with a mean weight z-score of −2.45, confirming consistent fetal growth impairment. Moreover, only 23.5% of reported cases reached term gestation, supporting the link between UPD(6)mat and prematurity. Failure to thrive (FTT) was reported in over half (52.4%) of the postnatal cases, further highlighting postnatal growth difficulties in this population. Monogenic disorders were identified in several cases, predominantly in isodisomic individuals. The most frequently affected gene was CYP21A2, involved in congenital adrenal hyperplasia due to 21-hydroxylase deficiency, observed in three independent patients. Other homozygous variants revealed through isodisomy included those causing 3M syndrome, molybdenum cofactor deficiency, Wiskott–Aldrich syndrome, and cone-rod dystrophies. Interestingly, trisomy 6 mosaicism was confirmed in three cases (12.5%), all involving heterodisomy or mixed disomy, and in one instance led to prenatal death at 23 weeks. This supports the theory that heterodisomy may result from trisomy rescue, with confined placental mosaicism (CPM) as a possible intermediate [10]. While trisomy 6 CPM remains rare, its presence raises concern for adverse outcomes, including IUGR and early pregnancy loss.

Our patient presented with features aligning with the emerging UPD(6)mat phenotype, including IUGR, prematurity, and SGA, but also displayed a unique sacrococcygeal teratoma, which to our knowledge has not been previously associated with UPD(6)mat. Moreover, the child exhibited a distinct neurobehavioral profile, with speech delay, autism spectrum features, and delayed motor development. Only one other report describes a similar presentation with motor delay and extremely limited vocabulary at 20 months of age, suggesting a possible shared neurodevelopmental signature in a subset of UPD(6)mat cases. Additional features in our case, such as pes planus and genu valgum, have also been previously reported in association with UPD(6)mat.

### 4.2. Possible Underlying Molecular/Cytogenetic Mechanisms of UPD6

Potential mechanisms underlying the UPD(6)mat phenotype include imprinting disturbances, cryptic monosomy and/or trisomy 6 mosaicism, and unmasking of autosomal recessive variants. Poke et al. suggested that homozygosity for recessive variants in 6q16.1–qter may cause IUGR, but Eggermann et al. found no shared isodisomic regions across cases, arguing against a common recessive gene [10,11]. Imprinting defects remain a plausible explanation, particularly as UPD is implicated in nine out of 13 known imprinting disorders. In UPD(6)pat, overexpression of PLAGL1 and HYMAI at 6q24 accounts for ~70% of transient neonatal diabetes mellitus (TNDM) cases [12]. While similar effects have not been confirmed in UPD(6)mat, this region remains of interest. Our review (Table S2, see Supplementary Materials) identified several candidate imprinted genes on chromosome 6, including ZAC/PLAGL1, involved in cell cycle arrest. In mice, loss of the paternal Zac1 allele leads to growth restriction, offering a potential mechanism for IUGR in UPD(6)mat [13]. Other known imprinted genes on chromosome 6 include MDGA1, MOCS1, C6orf47, RNF144B, CD83, FAM50B, AIM1, LIN28B, PHACTR2, HYMAI, SLC22A2, SLC22A3, PLG, KIF25, IGF2R, PXDC1, and WDR27 [14,15]. FAM50B methylation, linked to children’s IQs, should also be considered when investigating the UPD(6)mat phenotype [16]. The role of imprinting in UPD(6)mat remains uncertain. Evidence is limited, largely derived from placental studies or unrelated disorders. No recurrent variant or methylation pattern has been identified. Further multi-tissue epigenetic and genomic analyses are needed to clarify its molecular basis.

### 4.3. Neoplasms in Uniparental Disomy

In tumor cells, loss of heterozygosity (LOH) may arise from isodisomy and present as copy-neutral LOH, contributing to tumorigenesis through inactivation of tumor suppressor genes [17]. Although somatic UPD is recognized in cancer, tumors are not typically considered part of the congenital UPD phenotype. Our case represents the first documented instance of a congenital tumor—sacrococcygeal teratoma—associated with UPD(6)mat. It remains unclear whether the teratoma observed in our patient is related to UPD(6)mat or represents a sporadic finding. The vast majority of sacrococcygeal teratomas are sporadic, with a marked female predominance (4:1), and a family history of twin gestation reported in approximately 10% of cases [18]. Nevertheless, aberrant imprinting in UPD may increase susceptibility to neoplasms, particularly via dysregulation of growth and tumor suppressor pathways. This mechanism is well illustrated in Beckwith–Wiedemann syndrome, where paternal UPD(11) leads to IGF2 overexpression and a substantially increased risk of embryonal tumors. Most reported UPD(6)mat cases have been described after 2006 and involve young children with limited clinical follow-up. As such, late-onset manifestations—including tumor development—may still be underrecognized. Given the rarity of UPD(6)mat and these uncertainties, long-term clinical surveillance is advisable to better define its full phenotypic spectrum and potential oncologic risks. Given these uncertainties and the rarity of UPD(6)mat, long-term surveillance is recommended to better define its phenotypic spectrum and identify potential oncologic risks.

### 4.4. Diagnosis and Management of UPD6 Cases

Recent advances in molecular diagnostics have transformed the early detection of imprinting disorders. Imprinting defects are suspected to be the primary cause of the matUPD6 phenotype, which presents with varied nonspecific clinical features, such as intrauterine growth restriction, low birth weight, preterm labor, postnatal growth impairment, feeding difficulties, and delayed motor and cognitive development. Despite existing descriptive guidelines, no standardized algorithm for managing UPD(6)mat has been developed [1].

We propose a diagnostic approach combining targeted and non-targeted molecular techniques. While microsatellite analysis (MSA) is a gold standard in diagnosing individuals with UPD, in practice, all isodisomy cases and some heterodisomies can be detected or suspected through chromosomal microarray (CMA) platforms with SNP probes [19,20]. Currently, cases of heterodisomy are mostly picked up through trio whole-exome sequencing approaches. When methylation abnormalities are suspected, Methylation-Specific Multiplex Ligation-Dependent Probe Amplification (MS-MLPA) offers additional insights, with multilocus MS-MLPA having the widest coverage [21]. Further investigations, such as NGS testing for CYP21A2 variants (often associated with UPD(6)mat) and DNA methylation analysis, are critical for identifying genes and assessing epigenetic dysregulation. Given the diverse presentations of UPD(6)mat, management requires a multidisciplinary team, including specialists in genetics, endocrinology, neurology, and oncology. Table 2 outlines recommended interventions and tests based on insights from reported cases. Clinical care should prioritize monitoring growth, neurodevelopment, motor skills, and early tumor detection, with regular follow-up, as symptoms may evolve. Although no consistent genetic variant or methylation pattern has been linked to UPD(6)mat, comprehensive genomic analysis and personalized care are essential for improving patient outcomes.

## 5. Conclusions

We describe a rare case of maternal uniparental disomy of chromosome 6 (UPD(6)mat) with a combination of clinical features, including growth restriction, prematurity, developmental delay, autism spectrum disorder, and a sacrococcygeal teratoma. While some of these findings—particularly the tumor—may be incidental, their coexistence highlights the broad and still poorly understood phenotypic spectrum of UPD(6)mat. The underlying cause of the clinical features observed in UPD(6)mat remains uncertain. The most plausible explanations include disturbances in genomic imprinting, as seen in UPD(6)pat, and cryptic trisomy mosaicism, particularly in heterodisomic cases where trisomy rescue may have occurred. Looking forward, future research should focus on high-resolution genetic and epigenetic studies to better understand the mechanisms behind this phenotype. Defining the core clinical features and associated risks will be essential for developing evidence-based diagnostic and follow-up protocols. These should include systematic growth monitoring, neurodevelopmental assessment, and, when appropriate, tumor surveillance. Finally, consistent and detailed reporting of new cases—aligned with current classification guidelines, such as those by Liehr—will be key to improving clinical understanding and management of this rare genetic condition.

## Figures and Tables

**Table 1:** Main characteristics of the patient and genetic testing results, described according to Liehr 2022 guidelines for uniparental disomy patients [1].

UPD Criteria	Our Case
Gender and age of patient	Female; 4 years and 10 months
Prenatal	Polyhydramnios [HP:0001561]; IUGR [HP:0001511]
Clinical phenotype	Delayed speech and language development [HP:0000750]; Autism spectrum disorder [HP:0000729]; Triangular face [HP:0000325]; Prominent forehead [HP:0011220]; Full lips [HP:0012471]; Reduced subcutaneous adipose tissue [HP:0003758]; Sacrococcygeal teratoma [HP:0030736]; Sleep disturbance [HP:0002360]; Teeth grinding [HP:0003763]; Feeding difficulties [HP:0011968]; Auditory hypersensitivity [HP:5200060]; Genu valgum [HP:0002857]; Pes planus [HP:0001763]; Short attention span [HP:0000736]
Type of disomy	Maternal isodisomy
Affected chromosome	Whole chromosome 6
Test performed	Whole exome sequencing (WES) in the proband, her mother, and her father (trio)
A mosaic present	No mosaic present (peripheral blood cells examined only)

Abbreviations: UPD, uniparental disomy; IUGR, intrauterine growth restriction; HP, Human Phenotype Ontology ID number [7].

**Table 2:** Evaluations following initial diagnosis, treatment of manifestations, and surveillance of UPD(6)mat patients.

System/Concern	Evaluation	Comment	Treatment (If Needed)	Surveillance [Frequency]
Pregnancy and Perinatal Period	Measurements of weight, length, head circumference at birth, and the age at birth	Note IUGR, low birth weight, preterm labor, and any events during pregnancy		If any delayed growth signs are present, monitor further growth closely using appropriate percentile charts for preterm infants—Fenton [as needed]
Constitutional	Measurements of weight, height, head circumference	Monitor further growth closely using appropriate percentile charts	Evaluate the need for recombinant human growth hormone (rhGH) therapy	Measurement of growth parameters on percentile charts, monitor weight and height increments [at each visit]
Development, Behavior, and Neurological	Assessment of development and speech-language skills	Brain MRI if significant developmental delay is observed; Monitor for any features characteristic of Autism spectrum disorder; Monitor neurobehavioral patterns of the child	Neuropsychological therapy, cognitive therapy, speech therapy; Evaluate the need for special education institutions	Monitor developmental progress and educational needs [at each visit]
Musculoskeletal, Orthopedic, and Motor Skills	Evaluation of muscle tension, gross motor skills; Assessment of knee and foot alignment	Monitor for conditions such as pes planus and genu valgum	Physical therapy and orthopedic intervention as needed	Assessment of reaching motor milestones [at each visit]
Feeding	Evaluation of feeding effectiveness and dysphagia	Ensure adequate nutrition and assess for feeding difficulties	Nutritional support and feeding therapy	Monitor [at each visit]
Neoplasia	Abdominal ultrasound	Screen for any masses or tumors	Oncologic care as needed	
Facial Features and Skin	Assessment of facial features, subcutaneous tissue, skin characteristics	Evaluation for facial dysmorphia, facial clefts, lack of subcutaneous fat tissue		
Renal	Renal function tests and renal ultrasound	Monitor for renal insufficiency and structural anomalies	Nephrologic care as needed	
Adrenal	Screening for congenital adrenal hyperplasia (CAH)		Hormone replacement therapy if indicated	
Cardiovascular	Echocardiography	To assess heart morphology and for patent ductus arteriosus	Cardiac care as needed	

## Data Availability

No new data were created or analyzed in this study. Data sharing is not applicable to this article.

## Supplementary Materials

The following supporting information can be downloaded at: https://www.mdpi.com/article/10.3390/genes16040434/s1, Table S1. Summary of reported cases of maternal uniparental disomy of chromosome 6 in the literature [10,11,22,23,24,25,26,27,28,29,30,31,32,33,34,35,36,37,38]; Table S2. Summary of parentally imprinted genes on chromosome 6 [13,14,16,39,40,41,42].

## Abbreviations

The following abbreviations are used in this manuscript:

UPD	Uniparental disomy
UPD(6)mat	Maternal uniparental disomy of chromosome 6
IUGR	Intrauterine growth restriction
ASD	Autism spectrum disorder
WES	Whole exome sequencing
Trio WES	Whole exome sequencing for the proband and both parents
MRI	Magnetic resonance imaging
EEG	Electroencephalography
ASRS	Autism Spectrum Rating Scales
LOH	Loss of heterozygosity
CPM	Confined placental mosaicism
CMA	Chromosomal microarray
MSA	Microsatellite analysis
MS-MLPA	Methylation-Specific Multiplex Ligation-Dependent Probe Amplification
NGS	Next-generation sequencing
TNDM	Transient neonatal diabetes mellitus
SNP	Single nucleotide polymorphism

## References

[1] Liehr T. (2022). Uniparental disomy is a chromosomic disorder in the first place. Mol. Cytogenet..

[2] Engel E. (1980). A new genetic concept: Uniparental disomy and its potential effect, isodisomy. Am. J. Med. Genet..

[3] Spence J., Perciaccante R., Greig G., Willard H., Ledbetter D., Hejtmancik J., Pollack M.S., Obrien W., Beaudet A. (1988). Uniparental disomy as a mechanism for human genetic disease. Am. J. Hum. Genet..

[4] Nakka P., Smith S.P., O’donnell-Luria A.H., McManus K.F., Mountain J.L., Ramachandran S., Sathirapongsasuti J.F., Agee M., Auton A., Bell R.K. (2019). Characterization of Prevalence and Health Consequences of Uniparental Disomy in Four Million Individuals from the General Population. Am. J. Hum. Genet..

[5] Del Gaudio D., Shinawi M., Astbury C., Tayeh M.K., Deak K.L., Raca G. (2020). Diagnostic testing for uniparental disomy: A points to consider statement from the American College of Medical Genetics and Genomics (ACMG). Genet. Med..

[6] Temple I.K., Mackay D.J.G., Adam M.P., Feldman J., Mirzaa G.M., Pagon R.A., Wallace S.E., Amemiya A. (1993). Diabetes Mellitus, 6q24-Related Transient Neonatal. GeneReviews^®^.

[7] Gargano M.A., Matentzoglu N., Coleman B., Addo-Lartey E.B., Anagnostopoulos A.V., Anderton J., Avillach P., Bagley A.M., Bakštein E., Balhoff J.P. (2024). The Human Phenotype Ontology in 2024: Phenotypes around the world. Nucleic Acids Res..

[8] Fenton T.R., Kim J.H. (2013). A systematic review and meta-analysis to revise the Fenton growth chart for preterm infants. BMC Pediatr..

[9] Bione S., Sala C., Manzini C., Arrigo G., Zuffardi O., Banfi S., Borsani G., Jonveaux P., Philippe C., Zuccotti M. (1998). A human homologue of the Drosophila melanogaster diaphanous gene is disrupted in a patient with premature ovarian failure: Evidence for conserved function in oogenesis and implications for human sterility. Am. J. Hum. Genet..

[10] Eggermann T., Oehl-Jaschkowitz B., Dicks S., Thomas W., Kanber D., Albrecht B., Begemann M., Kurth I., Beygo J., Buiting K. (2017). The maternal uniparental disomy of chromosome 6 (upd(6)mat) “phenotype”: Result of placental trisomy 6 mosaicism?. Mol. Genet. Genom. Med..

[11] Poke G., Doody M., Prado J., Gattas M. (2013). Segmental Maternal UPD6 with Prenatal Growth Restriction. Mol. Syndromol..

[12] Docherty L.E., Kabwama S., Lehmann A., Hawke E., Harrison L., Flanagan S.E., Ellard S., Hattersley A.T., Shield J.P.H., Ennis S. (2013). Clinical presentation of 6q24 transient neonatal diabetes mellitus (6q24 TNDM) and genotype-phenotype correlation in an international cohort of patients. Diabetologia.

[13] Varrault A., Gueydan C., Delalbre A., Bellmann A., Houssami S., Aknin C., Severac D., Chotard L., Kahli M., Le Digarcher A. (2006). Zac1 regulates an imprinted gene network critically involved in the control of embryonic growth. Dev. Cell.

[14] Court F., Tayama C., Romanelli V., Martin-Trujillo A., Iglesias-Platas I., Okamura K., Sugahara N., Simón C., Moore H., Harness J.V. (2014). Genome-wide parent-of-origin DNA methylation analysis reveals the intricacies of human imprinting and suggests a germline methylation-independent mechanism of establishment. Genome Res..

[15] Jadhav B., Monajemi R., Gagalova K.K., Ho D., Draisma H.H., van de Wiel M.A., Franke L., Heijmans B.T., van Meurs J., Jansen R. (2019). RNA-Seq in 296 phased trios provides a high-resolution map of genomic imprinting. BMC Biol..

[16] Wan C., Ma H., Liu J., Liu F., Liu J., Dong G., Zeng X., Li D., Yu Z., Wang X. (2024). Quantitative relationships of FAM50B and PTCHD3 methylation with reduced intelligence quotients in school aged children exposed to lead: Evidence from epidemiological and in vitro studies. Sci. Total Environ..

[17] Makishima H., Maciejewski J.P. (2011). Pathogenesis and consequences of uniparental disomy in cancer. Clin. Cancer Res..

[18] Barksdale E.M., Obokhare I. (2009). Teratomas in infants and children. Curr. Opin Pediatr..

[19] Kearney H.M., Kearney J.B., Conlin L.K. (2011). Diagnostic implications of excessive homozygosity detected by SNP-Based microarrays: Consanguinity, uniparental disomy, and recessive single-gene mutations. Clin. Lab. Med..

[20] Hoppman N., Rumilla K., Lauer E., Kearney H., Thorland E. (2018). Patterns of homozygosity in patients with uniparental disomy: Detection rate and suggested reporting thresholds for SNP microarrays. Genet. Med..

[21] Bilo L., Ochoa E., Lee S., Dey D., Kurth I., Kraft F., Rodger F., Docquier F., Toribio A., Bottolo L. (2023). Molecular characterisation of 36 multilocus imprinting disturbance (MLID) patients: A comprehensive approach. Clin. Epigenetics.

[22] van den Berg-Loonen E.M., Savelkoul P., van Hooff H., van Eede P., Riesewijk A., Geraedts J. (1996). Uniparental maternal disomy 6 in a renal transplant patient. Hum. Immunol..

[23] Spiro R.P., Christian S.L., Ledbetter D.H., New M.I., Wilson R.C., Roizen N., Rosenfield R.L. (1999). Intrauterine growth retardation associated with maternal uniparental disomy for chromosome 6 unmasked by congenital adrenal hyperplasia. Pediatr Res..

[24] Cockwell A.E., Baker S.J., Connarty M., Moore I.E., Crolla J.A. (2006). Mosaic trisomy 6 and maternal uniparental disomy 6 in a 23-week gestation fetus with atrioventricular septal defect. Am. J. Med. Genet..

[25] Parker E.A., Hovanes K., Germak J., Porter F., Merke D.P. (2006). Maternal 21-hydroxylase deficiency and uniparental isodisomy of chromosome 6 and X results in a child with 21-hydroxylase deficiency and Klinefelter syndrome. Am J Med Genet A..

[26] Gümüş H., Ghesquiere S., Per H., Kondolot M., Ichida K., Poyrazoğlu G., Kumandaş S., Engelen J., Dundar M., Cağlayan A.O. (2010). Maternal uniparental isodisomy is responsible for serious molybdenum cofactor deficiency. Dev. Med. Child Neurol..

[27] Salahshourifar I., Halim A.S., Sulaiman W.A., Zilfalil B.A. (2010). Maternal uniparental heterodisomy of chromosome 6 in a boy with an isolated cleft lip and palate. Am. J. Med. Genet A.

[28] Sasaki K., Okamoto N., Kosaki K., Yorifuji T., Shimokawa O., Mishima H., Yoshiura K.I., Harada N. (2011). Maternal uniparental isodisomy and heterodisomy on chromosome 6 encompassing a CUL7 gene mutation causing 3M syndrome. Clin. Genet..

[29] Begemann M., Spengler S., Gogiel M., Grasshoff U., Bonin M., Betz R.C., Dufke A., Spier I., Eggermann T. (2012). Clinical significance of copy number variations in the 11p15.5 imprinting control regions: New cases and review of the literature. J. Med. Genet..

[30] Roosing S., van den Born L.I., Hoyng C.B., Thiadens A.A., de Baere E., Collin R.W., Koenekoop R.K., Leroy B.P., van Moll-Ramirez N., Venselaar H. (2013). Maternal uniparental isodisomy of chromosome 6 reveals a TULP1 mutation as a novel cause of cone dysfunction. Ophthalmology.

[31] Takimoto T., Takada H., Ishimura M., Kirino M., Hata K., Ohara O., Hara T. (2015). Wiskott-Aldrich Syndrome in a Girl Caused by Heterozygous WASP Mutation and Extremely Skewed X-Chromosome Inactivation: A Novel Association with Maternal Uniparental Isodisomy 6. Neonatology.

[32] Lazier J., Martin N., Stavropoulos J.D., Chitayat D. (2016). Maternal uniparental disomy for chromosome 6 in a patient with IUGR, ambiguous genitalia, and persistent mullerian structures. American Journal of Medical Genetics, Part A..

[33] Leung W.C., Lau W.L., Lo T.K., Lau T.K., Lam Y.Y., Kan A., Chan K., Lau E.T., Tang M.H. (2017). Two IUGR foetuses with maternal uniparental disomy of chromosome 6 or UPD(6)mat. J. Obstet. Gynaecol..

[34] Kerr E.R., Stuhlmiller G.M., Maha G.C., Ladd M.A., Mikhail F.M., Koester R.P., Hurst A.C. (2018). Maternal uniparental isodisomy for chromosome 6 discovered by paternity testing: A case report. Mol. Cytogenet..

[35] Souzeau E., Thompson J.A., McLaren T.L., De Roach J.N., Barnett C.P., Lamey T.M., Craig J.E. (2018). Maternal uniparental isodisomy of chromosome 6 unmasks a novel variant in TULP1 in a patient with early onset retinal dystrophy. Mol. Vis..

[36] Zhang P., Ying W., Wu B., Liu R., Wang H., Wang X., Lu Y. (2021). Complete IFN-γR1 Deficiency in a Boy Due to UPD(6)mat with IFNGR1 Novel Splicing Variant. J. Clin. Immunol..

[37] Jiang Y., Xiao Y.X., Xiong J.J., Zhang V.W., Dong C., Xu L., Liu F. (2024). Maternal uniparental disomy for chromosome 6 in 2 prenatal cases with IUGR: Case report and literature review. Mol. Cytogenet..

[38] Li J.W., Qian Y.J., Mao S.J., Chao Y.Q., Qin Y.F., Hu C.X., Li Z.L., Zou C.C. (2024). Clinical features associated with maternal uniparental disomy for chromosome 6. Mol. Cytogenet..

[39] Harris L.K., Pantham P., Yong H.E.J., Pratt A., Borg A.J., Crocker I., Westwood M., Aplin J., Kalionis B., Murthi P. (2019). The role of insulin-like growth factor 2 receptor-mediated homeobox gene expression in human placental apoptosis, and its implications in idiopathic fetal growth restriction. Mol. Hum. Reprod..

[40] Bradfield J.P., Qu H.Q., Wang K., Zhang H., Sleiman P.M., Kim C.E., Mentch F.D., Qiu H., Glessner J.T., Thomas K.A. (2011). A genome-wide meta-analysis of six type 1 diabetes cohorts identifies multiple associated loci. PLoS Genet..

[41] Xu Y., Goodyer C.G., Deal C., Polychronakos C. (1993). Functional polymorphism in the parental imprinting of the human IGF2R gene. Biochem. Biophys. Res. Commun..

[42] Mozaffari S.V., Stein M.M., Magnaye K.M., Nicolae D.L., Ober C. (2018). Parent of origin gene expression in a founder population identifies two new candidate imprinted genes at known imprinted regions. PLoS ONE.

