# Supplementary material for: Maternal Uniparental Isodisomy of Chromosome 6: A Novel Case of Teratoma and Autism Spectrum Disorder with a Diagnostic and Management Framework
Source: Genes (Basel). 2025 Apr 5;16(4):434. doi: 10.3390/genes16040434 (PMC12026494; doi:10.3390/genes16040434)
Supplement: Supplementary file 1 [file genes-16-00434-s001.zip › genes-3522201-supplementary-Table S1.pdf]

Table S1. Summary of reported cases of maternal uniparental disomy of chromosome 6 in the literature.

First Author, Year	Sex	UPD Type	Monogenic Mutation	Variant Consequences in a Patient	IUGR (HP:0001511)	Other Prenatal Findings	Gestational Age at birth (whole completed weeks)	Birth Weight (g)	Weight z-score	SGA, AGA OR LGA	Failure to Thrive (FTT)	Postnatal Phenotype other than correlating to the mutation
Our case	F	Isodisomy	DIAPH2 c.881T>A p.(Leu294His), heterozygous	NR	Yes	Polyhydramnios HP:0001561;	35	1450	-2,7	SGA	Yes	Sacrococcygeal teratoma HP:0030736; Delayed speech and language development HP:0000750; Autism Spectrum Disorder HP:0000717; Triangular face HP:0000325; Prominent forehead HP:0011220; Full lips HP:0012471; Reduced subcutaneous adipose tissue HP:0003758; Genu valgum HP:0002857; Pes planus HP:0001763;
Li, J. W., et al. (2024).	M	Hetero/Isodisomy	Not detected	NR	Yes	NR	32	1300	-1,5	SGA	Yes	Gastrostomy tube feeding in infancy HP:0011471; Allergic rhinitis HP:0003193; High palate HP:0000218;
Li, J. W., et al. (2024).	F	Isodisomy	SCUBE3 c.2330T > C (p. Phe-777Ser), homozygous	Short stature HP:0004322;	Yes	NR	36	1200	-3,9	SGA	Yes	none
Jiang, Y., et al. (2024).	M	Hetero/Isodisomy	Not detected	NR	Yes	NR	termination of pregnancy	no data	no data	no data	NR	NR
Jiang, Y., et al. (2024).	F	Hetero/Isodisomy	Not detected	NR	Yes	Fetal abdominal cyst HP:4000139	termination of pregnancy	no data	no data	no data	NR	NR
Zhang, P., et al. (2021).	M	Isodisomy	IFNGR1 c.861+2T>A, homozygous	AR complete IFN-γR1 deficiency	Yes	NR	40	2450	-2,8	SGA	No	none
Souzeau, E., et al. (2018).	F	Isodisomy	TULP1 c.524dupC, p.(Pro176ThrfsTer7), homozygous; RP1 (c.1267_1656delinsA, p.(Ala423AsnfsTer11)), heterozygous	Rod-cone dystrophy HP:0000510	Yes	Oligohydramnios HP:0001562;	37	1640	-3,3	SGA	No	none
Kerr, E. R., et al. (2018).	F	Isodisomy	Not detected	NR	No	Nuchal cord HP:0012498	32	1304	-1,3	SGA	No	Intraventricular hemorrhage HP:0030746; Preauricular pit HP:0004467;
Leung, W. C., et al. (2017).	F	Hetero/Isodisomy	mosaic placenta 47,XX, +6[12]/46,XX[19]	Confined placental mosaicism	Yes	Oligohydramnios HP:0001562;	34	1115	-3	SGA	No	none
Leung, W. C., et al. (2017).	F	Heterodisomy	mosaic placenta 47,XX, +6[14]/46,XX[16]	Confined placental mosaicism	Yes	NR	32	890	-2,6	SGA	No	none
Eggermann, T., et al. (2017).	F	Hetero/Isodisomy	46,XX (FISH: 98/100 normal, 2x monosomy 6)	NR	Yes	Bradycardia HP:0001662	27	650	-1,4	SGA	Yes	Clinodactyly of the 5th finger HP:0004209; Restlessness HP:0000711; Macrotia HP:0000400; Triangular face HP:0000325; Frontal bossing HP:0002007; Mandibular prognathia HP:0000303
Eggermann, T., et al. (2017).	M	Hetero/Isodisomy	CYP21A2: exon 1–8 deletion, homozygous	Adrenal hyperplasia, congenital, due to 21-hydroxylase deficiency OMIM:201910;	Yes	Oligohydramnios HP:0001562;	30	1100	-1,1	AGA	Yes	Short palpebral fissure HP:0012745; Macrotia HP:0000400; Clinodactyly of the 5th finger HP:0004209; Pes valgus HP:0008081; Pes planus HP:0001763; Macrotia HP:0000400
Lazier, J., et al. (2016).	M	Isodisomy	Not detected	NR	Yes	Placental dysfunction	28	680	-1,7	SGA	Yes	Ambiguous genitalia, male HP:0000033; Persistent Müllerian duct syndrome ORPHA:2856; Camptodactyly of finger HP:0100490; Perineal hypospadias HP:000005; Inguinal hernia HP:0000023; Patent ductus arteriosus HP:0001643; Thick septum pellucidum HP:0007375;
Takada, H., et al. (2014).	F	Isodisomy	WASP c.1276_1285 del_GCCCCTG GTG: p.Ala426GlyfsX15, heterozygous	Wiskott-Aldrich syndrome OMIM: 301000;	Yes	NR	29	641	-2	SGA	No	none
Roosing, S., et al. (2013).	M	Isodisomy	TULP1 c.1258C A; p.Arg420Ser, homozygous	Cone dystrophy HP:0008020;	Yes	NR	40	no data	no data	no data	No	none
Poke, G., et al. (2013).	F	Isodisomy	Not detected	NR	Yes	Decreased fetal movement HP:0001558; Placental insufficiency ORPHA:439167; Abnormal placenta morphology HP:0100767;	35	1200	-3,5	SGA	Yes	Dysphagia HP:0002015; Gastroesophageal reflux HP:0002020; Global developmental delay HP:0001263
Begemann, M., et al., (2012).	F	Heterodisomy	Not detected	NR	Yes	Decreased fetal movement HP:0001558; Oligohydramnios HP:0001562;	34	1030	-3,2	SGA	Yes	Midface retrusion HP:0011800; Prominent forehead HP:0011220; Relative macrocephaly HP:0004482; Hypotonia HP:0001252; Motor delay HP:0001270; Umbilical hernia HP:0001537;
Sasaki, K., et al. (2011).	M	Hetero/Isodisomy	CUL7: c.2975G > C (p.R992P), homozygous	3M-syndrome OMIM: 273750;	Yes	NR	36	1000	-4,6	SGA	Yes	Mild mental retardation HP:0001256; Inguinal hernia HP:0000023; Hydrocele testis HP:0000034; Ventriculomegaly HP:0002119; Feeding difficulties in infancy HP:0008872; Hypotonia HP:0001252; Hypospadias HP:0000047; Triangular face HP:0000325; Malar flattening HP:0000272; Epicanthus HP:0000286; Short nose HP:0003196; Anteverted nares HP:0000463; Full lips HP:0012471; Long philtrum HP:0000343; Hypermobility of toe joints HP:0010510; Slender long bone HP:0003100; Pointed chin HP:0000307;
Salahshourifar, I., et al. (2010).	M	Heterodisomy	Not detected	NR	No	Maternal hypertension HP:0008071	40	3700	0,2	AGA	No	Orofacial cleft HP:0000202;
Gümüş, H., et al. (2010).	M	Isodisomy	MOCS1: c.217C>T (p.Arg73Trp), homozygous	Molybdenum cofactor deficiency, OMIM:252150;	No	Dandy-Walker malformation HP:0001305;	40	3750	0,3	AGA	No	Triangular face HP:0000325; Anteverted nares HP:0000463; Microcephaly HP:0000252; Motor delay HP:0001270;
Parker, E. A., et al. (2006).	M	Isodisomy	48,XXY, mar[30]/ 47,XXY[20], CYP21A2 whole gene deletion, homozygous, UPD X(mat)	Klinefelter syndrome; Adrenal hyperplasia, congenital, due to 21-hydroxylase deficiency OMIM:201910;	Yes	Oligohydramnios HP:0001562	36	2060	-1,8	SGA	Yes	Persistent pulmonary hypertension of the newborn HP: 0002092; Microcephaly HP:0000252; Patent ductus arteriosus HP:0001643;

Cockwell, A. E., et al. (2006).	M	Heterodisomy	mos46,XY[13]/47,XY, + 6[12]	NR	NR	Prenatal death HP:0034241	23	no data	no data	no data	NR	Prenatal death HP:0034241; Atrioventricular canal defect HP:0006695; Omphalocele HP:0001539;
Spiro, R. P., et al. (1999).	F	Isodisomy	CYP21A2: (p.I172N), homozygous	Adrenal hyperplasia, congenital, due to 21-hydroxylase deficiency OMIM:201910;	Yes	NR	37	1410	-3,9	SGA	No	Motor delay HP:0001270; Chronic otitis media HP:0000389;
van den Berg-Loonen (1996).	M	Isodisomy	Not detected	NR	Yes	NR	40	1500	-5,3	SGA	Yes	Sarcoidosis ORPHA:797; Renal insufficiency HP:0000083;

References

Begemann, M., Spengler, S., Gogiel, M., Grasshoff, U., Bonin, M., Betz, R. C., Dufke, A., Spier, I., & Eggermann, T. (2012). Clinical significance of copy number variations in the 11p15.5 imprinting control regions: new cases and review of the literature. J Med Genet, 49(9), 547-553. https://doi.org/10.1136/jmedgenet-2012-100967

Cockwell, A. E., Baker, S. J., Connarty, M., Moore, I. E., & Crolla, J. A. (2006). Mosaic trisomy 6 and maternal uniparental disomy 6 in a 23-week gestation fetus with atrioventricular septal defect [Article]. American Journal of Medical Genetics, 140 A(6), 624-627. https://doi.org/10.1002/ajmg.a.31129

Eggermann, T., Oehl-Jaschkowitz, B., Dicks, S., Thomas, W., Kanber, D., Albrecht, B., Begemann, M., Kurth, I., Beygo, J., & Buiting, K. (2017). The maternal uniparental disomy of chromosome 6 (upd(6)mat) "phenotype": result of placental trisomy 6 mosaicism? Mol Genet Genomic Med, 5(6), 668-677. https://doi.org/10.1002/mgg3.324

Gümüş, H., Ghesquiere, S., Per, H., Kondolot, M., Ichida, K., Poyrazoğlu, G., Kumandaş, S., Engelen, J., Dundar, M., & Cağlayan, A. O. (2010). Maternal uniparental isodisomy is responsible for serious molybdenum cofactor deficiency. Dev Med Child Neurol, 52(9), 868-872. https://doi.org/10.1111/j.1469-8749.2010.03724.x

Jiang, Y., Xiao, Y. X., Xiong, J. J., Zhang, V. W., Dong, C., Xu, L., & Liu, F. (2024). Maternal uniparental disomy for chromosome 6 in 2 prenatal cases with IUGR: case report and literature review. Mol Cytogenet, 17(1), 1. https://doi.org/10.1186/s13039-023-00670-0

Kerr, E. R., Stuhlmiller, G. M., Maha, G. C., Ladd, M. A., Mikhail, F. M., Koester, R. P., & Hurst, A. C. E. (2018). Maternal uniparental isodisomy for chromosome 6 discovered by paternity testing: a case report. Mol Cytogenet, 11, 60. https://doi.org/10.1186/s13039-018-0411-3

Lazier, J., Martin, N., Stavropoulos, J. D., & Chitayat, D. (2016). Maternal uniparental disomy for chromosome 6 in a patient with IUGR, ambiguous genitalia, and persistent mullerian structures [Article]. American Journal of Medical Genetics, Part A, 170(12), 3227-3230. https://doi.org/10.1002/ajmg.a.37876

Leung, W. C., Lau, W. L., Lo, T. K., Lau, T. K., Lam, Y. Y., Kan, A., Chan, K., Lau, E. T., & Tang, M. H. (2017). Two IUGR foetuses with maternal uniparental disomy of chromosome 6 or UPD(6)mat [Article]. Journal of Obstetrics and Gynaecology, 37(1), 113-115. https://doi.org/10.1080/01443615.2016.1242558

Li, J. W., Qian, Y. J., Mao, S. J., Chao, Y. Q., Qin, Y. F., Hu, C. X., Li, Z. L., & Zou, C. C. (2024). Clinical features associated with maternal uniparental disomy for chromosome 6. Mol Cytogenet, 17(1), 18. https://doi.org/10.1186/s13039-024-00688-y

Parker, E. A., Hovanes, K., Germak, J., Porter, F., & Merke, D. P. (2006). Maternal 21-hydroxylase deficiency and uniparental isodisomy of chromosome 6 and X results in a child with 21-hydroxylase deficiency and Klinefelter syndrome. Am J Med Genet A, 140(20), 2236-2240. https://doi.org/10.1002/ajmg.a.31408

Poke, G., Doody, M., Prado, J., & Gattas, M. (2013). Segmental Maternal UPD6 with Prenatal Growth Restriction. Mol Syndromol, 3(6), 270-273. https://doi.org/10.1159/000345168

Roosing, S., van den Born, L. I., Hoyng, C. B., Thiadens, A. A., de Baere, E., Collin, R. W., Koenekoop, R. K., Leroy, B. P., van Moll-Ramirez, N., Venselaar, H., Riemslag, F. C., Cremers, F. P., Klaver, C. C., & den Hollander, A. I. (2013). Maternal uniparental isodisomy of chromosome 6 reveals a TULP1 mutation as a novel cause of cone dysfunction. Ophthalmology, 120(6), 1239-1246. https://doi.org/10.1016/j.ophtha.2012.12.005

Salahshourifar, I., Halim, A. S., Sulaiman, W. A., & Zilfalil, B. A. (2010). Maternal uniparental heterodisomy of chromosome 6 in a boy with an isolated cleft lip and palate. Am J Med Genet A, 152a(7), 1818-1821. https://doi.org/10.1002/ajmg.a.33526

Sasaki, K., Okamoto, N., Kosaki, K., Yorifuji, T., Shimokawa, O., Mishima, H., Yoshiura, K. I., & Harada, N. (2011). Maternal uniparental isodisomy and heterodisomy on chromosome 6 encompassing a CUL7 gene mutation causing 3M syndrome. Clin Genet, 80(5), 478-483. https://doi.org/10.1111/j.1399-0004.2010.01599.x

Souzeau, E., Thompson, J. A., McLaren, T. L., De Roach, J. N., Barnett, C. P., Lamey, T. M., & Craig, J. E. (2018). Maternal uniparental isodisomy of chromosome 6 unmasks a novel variant in TULP1 in a patient with early onset retinal dystrophy. Mol Vis, 24, 478-484. https://pmc.ncbi.nlm.nih.gov/articles/PMC6066270/pdf/mv-v24-478.pdf

Spiro, R. P., Christian, S. L., Ledbetter, D. H., New, M. I., Wilson, R. C., Roizen, N., & Rosenfield, R. L. (1999). Intrauterine growth retardation associated with maternal uniparental disomy for chromosome 6 unmasked by congenital adrenal hyperplasia. Pediatr Res, 46(5), 510-513. https://doi.org/10.1203/00006450-199911000-00004

Takimoto, T., Takada, H., Ishimura, M., Kirino, M., Hata, K., Ohara, O., Morio, T., & Hara, T. (2015). Wiskott-Aldrich Syndrome in a Girl Caused by Heterozygous WASP Mutation and Extremely Skewed X-Chromosome Inactivation: A Novel Association with Maternal Uniparental Isodisomy 6. Neonatology, 107(3), 185-190. https://doi.org/10.1159/000370059

van den Berg-Loonen, E. M., Savelkoul, P., van Hooff, H., van Eede, P., Riesewijk, A., & Geraedts, J. (1996). Uniparental maternal disomy 6 in a renal transplant patient. Human Immunology, 45(1), 46-51. https://doi.org/https://doi.org/10.1016/0198-8859(95)00148-4

Zhang, P., Ying, W., Wu, B., Liu, R., Wang, H., Wang, X., & Lu, Y. (2021). Complete IFN-γR1 Deficiency in a Boy Due to UPD(6)mat with IFNGR1 Novel Splicing Variant [Letter]. Journal of Clinical Immunology, 41(4), 834-836. https://doi.org/10.1007/s10875-021-00970-3
