# Supplementary material for: Maternal Uniparental Isodisomy of Chromosome 6: A Novel Case of Teratoma and Autism Spectrum Disorder with a Diagnostic and Management Framework
Source: Genes (Basel). 2025 Apr 5;16(4):434. doi: 10.3390/genes16040434 (PMC12026494; doi:10.3390/genes16040434)
Supplement: Supplementary file 1 [file genes-16-00434-s001.zip › genes-3522201-supplementary-Table S2.pdf]

Table S2. Summary of parentally imprinted genes on chromosome 6.

Gene	Cytogenic location	Function	Expressed allele according to (Tucci et al., 2019)	Associated diseases according to MalaCards (https://www.malacards.org)	Other
MDGA1 OMIM: 609626 GC06M093942	6p21.2	Encodes a glycosylphosphatidylinositol (GPI)-anchored cell surface glycoprotein that is expressed predominantly in the developing	Not known	Bipolar Disorder; Osgood-Schlatter's Disease; Schizophrenia;	Plays a role in neuronal migration and synaptic formation.
MOCS1 OMIM: 603707	6p21.2	Encodes a protein involved in molybdenum cofactor biosynthesis	Paternal	Molybdenum Cofactor Deficiency;	-
C6orf47 GC06M093787	6p21.33	No data	Paternal	No data	-
RNF144B OMIM: 618869	6p22.3	Encodes an E3 ubiquitin-protein ligase involved in regulation of homeostasis in stratified	Not known	Chordoma;	-
CD83 OMIM: 604534	6p23	Encodes a single-pass type I membrane protein and member of the immunoglobulin superfamily	Not known	Neonatal Infective Mastitis; Plague;	May play a significant role in antigen presentation or the cellular interactions that follow lymphocyte activation.
FAM50B OMIM:614686	6p25.2	Contains an intronless open reading frame that arose from ancestral retroposition	Paternal	Armfield Syndrome; Temple Syndrome;	Quantitative linear relationship between children's IQs and FAM50B/PTCHD3 DNA methylation levels, and between FAM50B DNA methylation and reactive oxygen species (ROS) production. (Wan et al., 2024)
AIM1 OMIM:601797	6q21	predicted to enable carbohydrate binding activity	Paternal	Melanoma; Atrial Septal Defect 4;	May function as suppressor of malignant melanoma.
LIN28B OMIM: 611044	6q21	Encodes a protein belonging to the lin-28 family, characterized by a cold-shock domain and CCHC zinc finger domains	Paternal	Neuroblastoma; Acrocephalopolysyndactyly Type III;	-
PHACTR2 OMIM: 608724	6q24	Predicted to enable actin binding activity	Maternal	Body Dysmorphic Disorder; Parkinson's Disease;	Biomarker of Alzheimer's disease.
HYMAI OMIM: 606546	6q24	Encodes a non-protein coding transcript	Paternal	Transient Neonatal Diabetes Mellitus;	Causative gene for transient neonatal diabetes mellitus;
ZAC/PLAGL1 OMIM: 603044	6q24.2	Encodes a C2H2-type zinc-finger protein involved in cell cycle arrest	Paternal	Transient Neonatal Diabetes Mellitus;	Maternal allele absence leads to normal development; paternal allele absence causes weight reduction. (Varrault et
SLC22A2 OMIM: 602608	6q25	Encodes a polyspecific organic cation transporter	Maternal	Systemic Primary Carnitine Deficiency; Type 1 Diabetes Mellitus 8;	Critical for elimination of many endogenous small organic cations and various drugs and toxins.
SLC22A3 OMIM: 604842	6q25	Encodes a polyspecific organic cation transporter	Maternal	Type 1 Diabetes Mellitus 8; Obsessive-Compulsive Disorder;	Critical for elimination of many endogenous small organic cations and various drugs and toxins.
PLG OMIM: 173350	6q26	Encodes plasminogen protein	Paternal	Plasminogen Deficiency Type I; Hereditary Angioedema Type 4;	-
KIF25 OMIM: 603815	6q27	Encodes a protein that is a member of the kinesin-like protein family	Not known	Thrombophlebitis Migrans; Teebi Hypertelorism Syndrome 1;	-
IGF2R OMIM: 147280	6q25.3	encodes a IGF2R receptor for IGF2 and other molecules, involved in lysosomal signaling, functions as tumor suppressor gene	Maternal in a minority (polymorphic trait), biallelic in most (Xu et al., 1993)	Hepatocellular Carcinoma;	IGF2R expression was significantly decreased in placentas from pregnancies complicated by idiopathic fetal growth restriction. (Harris et al., 2019)
PXDC1 GC06M003723	6p25.2	Predicted to enable phosphatidylinositol binding activity	Mostly paternal (Mozaffari et al., 2018)	No data	-
WDR27 GC06M169474	6q27	Encodes a scaffold protein with multiple WD repeat domains and is ubiquitously expressed in the human body	Paternal (Court et al., 2014)	Cranioectodermal Dysplasia 2;	Affected pathways in Alzheimer's disease when WDR27 genes are silenced. (Bradfield et al., 2011)

References

1. Wan, C., et al., Quantitative relationships of FAM50B and PTCHD3 methylation with reduced intelligence quotients in school aged children exposed to lead: Evidence from epidemiological and in vitro studies. Science of The Total Environment, 2024. 907: p. 167976.
2. Varrault, A., et al., Zac1 regulates an imprinted gene network critically involved in the control of embryonic growth. Dev Cell, 2006. 11(5): p. 711-22.
3. Harris, L.K., et al., The role of insulin-like growth factor 2 receptor-mediated homeobox gene expression in human placental apoptosis, and its implications in idiopathic fetal growth restriction. Mol Hum Reprod, 2019. 25(9): p. 572-585.
4. Bradfield, J.P., et al., A genome-wide meta-analysis of six type 1 diabetes cohorts identifies multiple associated loci. PLoS Genet, 2011. 7(9): p. e1002293.
5. Xu, Y., et al., Functional polymorphism in the parental imprinting of the human IGF2R gene. Biochem Biophys Res Commun, 1993. 197(2): p. 747-54.
6. Mozaffari, S.V., et al., Parent of origin gene expression in a founder population identifies two new candidate imprinted genes at known imprinted regions. PLoS One, 2018. 13(9): p. e0203906.
7. Court, F., et al., Genome-wide parent-of-origin DNA methylation analysis reveals the intricacies of human imprinting and suggests a germline methylation-independent mechanism of establishment. Genome Res, 2014. 24(4): p. 554-69.
